# A novel approach to correcting attribution of *Clostridioides difficile* in a healthcare setting

**DOI:** 10.1017/ash.2023.516

**Published:** 2023-12-20

**Authors:** Hunter Doyle, Abby L. Valek, Theresa Murillo, Ashley M. Ayres, Julie Slaughter, Madeline L. Berg, Graham M. Snyder

**Affiliations:** 1 Department of Epidemiology, University of Pittsburgh School of Public Health, Pittsburgh, PA, USA; 2 Department of Infection Prevention and Control, UPMC Presbyterian/Shadyside, Pittsburgh, PA, USA; 3 Department of Infection Prevention and Control, UPMC Senior Communities, Pittsburgh, PA, USA; 4 Division of Infectious Diseases, Department of Medicine, University of Pittsburgh School of Medicine, Pittsburgh, PA, USA

## Abstract

**Objective:**

To describe a novel attribution metric estimating the causal source location of healthcare-associated *Clostridioides difficile* and compare it with the current US National Healthcare Safety Network (NHSN) surveillance reporting standard.

**Design:**

Quality improvement study.

**Setting:**

Two acute care facilities.

**Methods:**

A novel attribution metric assigned days of attribution to locations where patients were located for 14 days before and the day of their *C. difficile* diagnosis. We correlated the NHSN-assigned unit attribution with the novel attribution measure and compared the proportion of attribution assigned to inpatient units.

**Results:**

During a 30-month period, there were 727 NHSN *C. difficile* healthcare-associated infections (HAIs) and 409 non-HAIs; the novel metric attributed 17,034 days. The correlation coefficients for NHSN and novel attributions among non-ICU units were 0.79 (95% CI, 0.76–0.82) and 0.74 (95% CI, 0.70–0.78) and among ICU units were 0.70 (95% CI, 0.63–0.76) and 0.69 (95% CI, 0.60–0.77) at facilities A and B, respectively. The distribution of difference in percent attribution showed higher inpatient unit attribution using NHSN measure than the novel attribution metric: 38% of ICU units and 15% of non-ICU units in facility A, and 20% of ICU units and 25% of non-ICU units in facility B had a median difference >0; no inpatient units showed a greater attribution using the novel attribution metric.

**Conclusion:**

The novel attribution metric shifts attribution from inpatient units to other settings and correlates modestly with NHSN methodology of attribution. If validated, the attribution metric may more accurately target *C. difficile* reduction efforts.

## Introduction

*Clostridioides difficile* infection is a burden to healthcare facilities, resulting in up to 300,000 healthcare-associated infections (HAIs) annually.^1^ Prior research has established patient-to-patient transmission in hospitals; however, whole genome sequencing-based studies have challenged traditional epidemiologic methods in accurately identifying transmission events. In population-based studies defining a putative source of *C. difficile* by identifying genetically related isolates and then characterizing epidemiological linkages, nearly half of patient infections had no apparent source.^2^ Transmission can occur between facilities^3,4^ and in community settings,^5^ which may contribute to incorrect attribution of HAIs. On a facility level, genomic methods have demonstrated that putative transmission by epidemiological methods is frequently incorrect and overestimates the likelihood of in-hospital transmission.^6–8^ Therefore, the location of diagnosis likely inadequately correlates with where acquisition occurred.

Furthermore, there is uncertainty surrounding incubation periods which may be longer than assumed using HAI surveillance methodology.^9,10^ The use of antibiotics increases the risk of *C. difficile* infection.^11,12^ When present, this “trigger” could occur days before infection.^13,14^ Hospitals use National Healthcare Safety Network (NHSN) guidelines to define a *C. difficile* HAI. A *C. difficile* sample collected on or after hospital day 3 is considered an HAI, while collection on day 1 and 2 is present on admission.^15^ This definition may not accurately reflect where the acquisition of the organism or antibiotic trigger for the development of symptomatic infection occurred.

Unlike other HAI, targets of zero or nearly zero *C. difficile* events are not viewed as achievable.^16^ Current prevention efforts such as contact precautions and hand washing are necessary but may not be sufficient to prevent such transmission.^17^ Antimicrobial stewardship has also been used to decrease healthcare-associated *C. difficile* during patient stays within facilities.^18^ Bundled efforts for HAI *C. difficile* result in as much as a 82.3% reduction.^19^ Targeting healthcare locations where transmission or antibiotic exposure is not contributing to *C. difficile* HAI may account for incomplete HAI reduction. More accurately identifying which care locations are associated with transmission or antibiotic exposure may improve *C. difficile* prevention efforts.

Improving the attribution of the transmission event and antibiotic trigger for *C. difficile* will allow more efficient and effective use of resources to prevent *C. difficile*-related harm. Genomic analysis of all isolates in a community will provide this information, but at the present is a costly resource not available in most facilities. An inexpensive and accessible method of assigning *C. difficile* attribution may be effective until low-cost high-throughput genomic analysis is widely available. We devised a novel *C. difficile* attribution measure that may direct *C. difficile* reduction efforts more accurately. The aim of this quality improvement initiative study was to describe the novel metric estimating the causal source location of healthcare-associated *C. difficile* colitis, and as an initial evaluation of the utility of the metric, to compare the attribution ascribed by the novel metric with the current US surveillance standard.

## Methods

### Study setting

Facility A and facility B are acute care hospitals of the UPMC healthcare system in Pittsburgh, Pennsylvania. Facility A is a level 1 regional resource trauma center that specializes in solid organ transplantation and has 695 beds, including 134 intensive care unit (ICU) beds. Facility B is a tertiary care hospital that specializes in oncology and has 520 beds, including 66 ICU beds.

This project underwent formal ethical review and was granted approval as a quality improvement study by the UPMC Quality Improvement Review Committee. Methods and results are reported in accordance with Strengthening the Reporting of Observational Studies in Epidemiology (STROBE) statement (Supplemental material 1).

The study facilities have established ordering guidelines with electronic health record decision support for *C. difficile*: testing is recommended only if the patient has ≥3 unformed stools within 24 hours that are not due to laxatives, tube feeds, bowel prep, or enemas, plus one of the following: antibiotic exposure within the last 60 days, fever >38°C, abdominal tenderness/cramping/distention, white blood cell count >10,000 white blood cells per mm^3^ within 24 hours of unformed stools, recent chemotherapy/immunosuppression, or history of *C. difficile* infection. The laboratory rejects stools that do not conform to the shape of the container.

Patients who test positive for *C. difficile* (regardless of whether the result represents colonization or infection) are placed in contact precautions (gown and gloves). Hand hygiene is performed using soap and water. Environmental cleaning is performed with a hypochlorite-based product. These precautions are applied for the duration of the index admission, regardless of symptom resolution, and may be discontinued during a prolonged admission or re-instituted on a subsequent admission on a case-by-case basis.

UPMC facilities perform *C. difficile* testing using a two-step testing method.^20^ First, an enzyme immunoassay (EIA) is performed that tests for glutamate dehydrogenase (GDH) and *C. difficile* toxin. If there is a discordant result (GDH detected, toxin not detected), a polymerase chain reaction (PCR) is performed to detect the toxin-producing gene. A negative PCR equates to a negative result and a positive result advises clinical correlation to determine *C. difficile* colonization versus infection. Both concordant GDH detected, and toxin detected results, as well as discordant GDH detected, and toxin not detected results with a positive PCR are interpreted as positive for NHSN reporting purposes. *C. difficile* HAIs are defined using NHSN guidelines.^15^ Patients who test positive for *C. difficile* with a sample collected on hospital day 1 or 2 are labeled as present on admission, and those collected after day 2 of their hospital stay are reported as HAI. The unit of attribution is defined as the earliest location the patient was on the day prior to sample collection.^15^ This “conventional” attribution is commonly interpreted as the source location for the infection when planning prevention interventions.

## Data sources and outcomes

The conventional attribution metric was generated using NHSN-defined *C. difficile* HAI and unit location from July 2019 to December 2021. Monthly HAI counts for each unit were divided by the total number of cases in the facility in that month (Box 1). The metric represents each unit’s accountability for the *C. difficile* HAI cases in that facility for the month.

The novel attribution metric was generated using all NHSN-reported positive *C. difficile* tests obtained from July 2019 to December 2021, including infections defined by NHSN as present on admission and HAI.^15^ A chart review was performed to determine where each patient with a positive test was located during the 14 days prior to and the day of positive sample collection. Locations were derived from hospital electronic health records and infection prevention surveillance software and included preadmission ambulatory care visits and care in skilled nursing facilities, in addition to inpatient units. Within each of the 15 days, multiple locations in a day were divided evenly among respective locations. For example, a patient presenting to an emergency department from home on the same day would have 0.5 days attributed to home and 0.5 days attributed to the emergency department. Locations were grouped into categories of non-facility attribution (community, other organization acute care facility, within-organization acute care facility, and non-acute care) and facility attribution (ICU and non-ICU unit types). Monthly attribution days for each location or location category were divided by total attribution days for the index test facility (number of cases times 15 days) to create the attribution metric (Box 1).

Box 1. Calculations for the conventional National Healthcare Safety Network *Clostridioides difficile* healthcare-associated infection (HAI) attribution and novel *Clostridioides difficile* attribution metrics.

**Conventional attribution for each unit, per month**


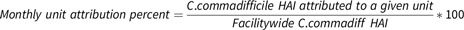

**Novel attribution calculation for each unit, per month**


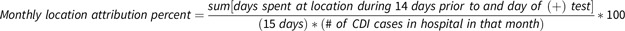

### Data visualization

**Figure 1.**
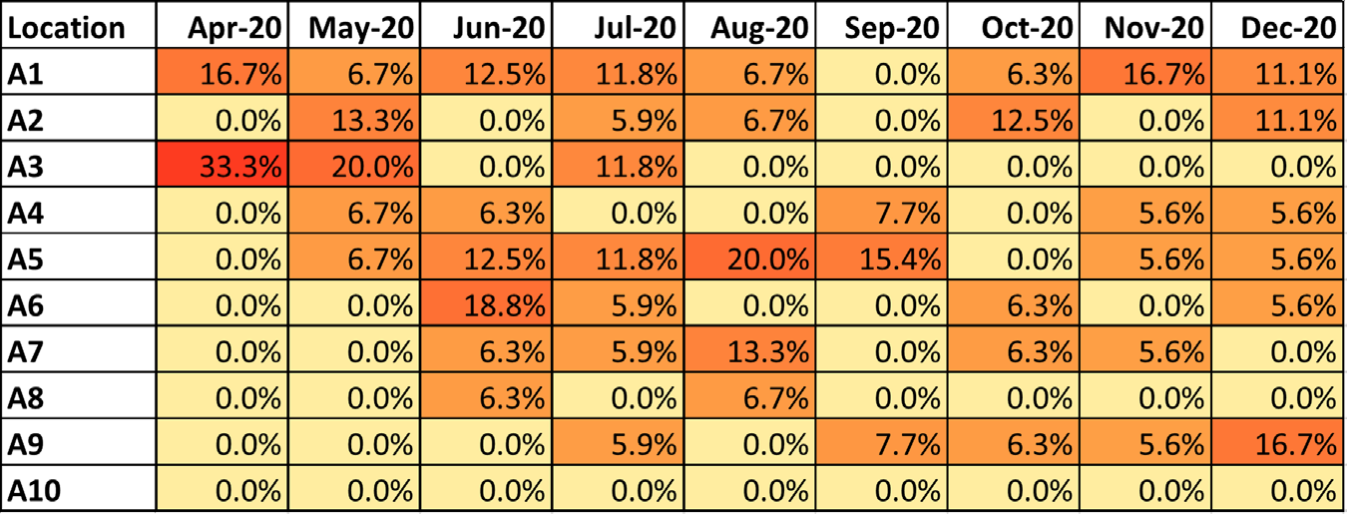
Representative heat map of the *Clostridioides difficile* conventional attribution metric in an acute care facility.

A heat map was created for each attribution method to visualize the unit-month attribution percent. The heat map is the primary tool to guide infection prevention interventions as part of quality improvement work. The heat map plots unit versus month with the intensity of color of each cell corresponding to the proportion of accountability ascribed to that unit within the facility for the month (Figure 1).

### Statistical methods

To evaluate the concordance between the conventional and novel methods, a correlation coefficient was calculated with each data point representing the conventional attribution percentage and the novel attribution percentage for each unit, monthly. These analyses, stratified by facility, were restricted to NHSN-defined locations. Because this analysis is not intended to demonstrate that the novel attribution measure is “correct,” only to observe how significant it changes the attribution of *C. difficile* disease, we hypothesized that the correlation coefficient would be neither very strong nor very weak and therefore performed no statistical tests of significance.^21^ As a sensitivity analysis, the attribution measures were re-calculated using attribution days aggregated by quarter rather than monthly.

We also calculated the monthly difference in *C. difficile* percent attribution (NHSN-defined attribution minus novel attribution measure) for each unit. These were visualized to describe the distribution of difference in percent attribution. We hypothesized that since the novel attribution metric would “reallocate” *C. difficile* attribution to non-NHSN-defined locations, including preadmission locations of care, units would consistently have a lower attribution using the novel metric compared to the conventional metric.

## Results

During the 30-month study period, there were 727 NHSN-adjudicated *C. difficile* HAI, 450 (61.9%) at facility A and 277 (38.1%) at facility B (Table 1). There were 409 *C. difficile* diagnoses that were non-HAI (204 [49.9%] facility A, 205 [50.1%] facility B); therefore, a total of 1136 *C. difficile* diagnoses attributed as HAI and non-HAI were used to calculate the novel attribution.

**Table 1.** Distribution of healthcare-associated *Clostridioides difficile* infections, by facility and unit type

Facility, location	Number of units	Attributed healthcare-associated infection
Facility A, non-ICU	27	308
Facility A, ICU	8	142
Facility A, Total	35	450
Facility B, non-ICU	16	229
Facility B, ICU	5	48
Facility B, Total	21	277
Combined, non-ICU	43	537
Combined, ICU	13	190
Combined, total	56	727

ICU, intensive care unit.

The novel metric attributed a total of 17034.1 days of *C. difficile* risk, including 9,791.4 days attributed to prehospitalization sources, and 7,242.7 days attributed to facility A and facility B locations (Table 2).

**Table 2.** Novel attribution measure for nosocomial and community attribution of *Clostridioides difficile*

Location type	Novel attribution measure – total number of days (%)	
	Facility A	Facility B
**Preadmission sources**		
Community	4181.2 (42.6%)	3582.0 (49.6%)
Other organization acute care facility	134.7 (1.4%)	82.8 (1.1%)
Within organization other acute care facility	455.4 (4.6%)	238.4 (3.3%)
Non-acute care facility	547.3 (5.6%)	569.6 (7.9%)
**Nosocomial attribution**		
Non-intensive care unit	2930.9 (29.9%)	2188.1 (30.3%)
Intensive care unit	1558.9 (15.9%)	564.7 (7.8%)
**Total**	9808.4	7225.7

**Figure 2.**
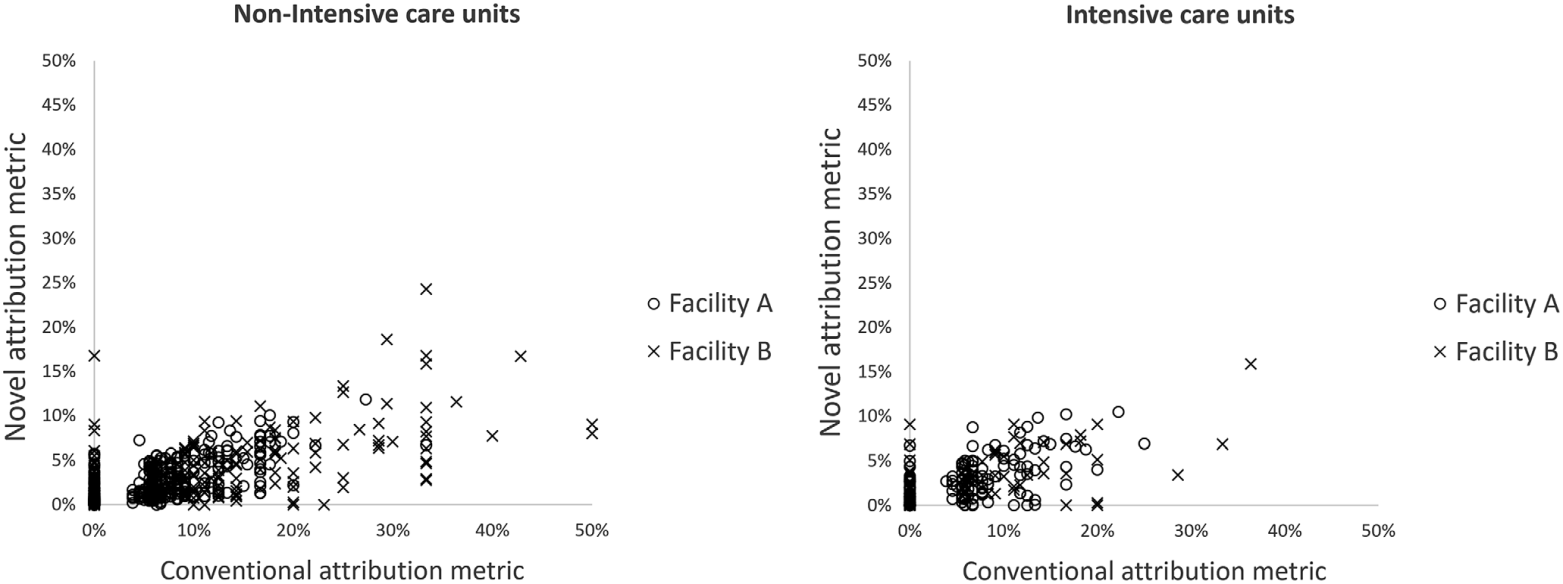
Frequency of the monthly *Clostridioides difficile* infections attributed to intensive care units and non-intensive care units at two study facilities, comparing conventional and novel attribution metrics.

Facility A had 35 NHSN-defined units (27 non-ICU, 8 ICU) which over 30 months of follow-up yielded 1050 unit-month attribution percent data points (810 non-ICU, 240 ICU). In facility B, 21 units (16 non-ICU, 5 ICU) yielded 630 data points (480 non-ICU, 150 ICU) (Figure 2). The correlation coefficients for non-ICU units were 0.79 (95% CI, 0.76–0.82) and 0.74 (95% CI, 0.70–0.78) and for ICU units were 0.70 (95% CI, 0.63–0.76) and 0.69 (95% CI, 0.60–0.77) at facilities A and B, respectively.

**Figure 3.**
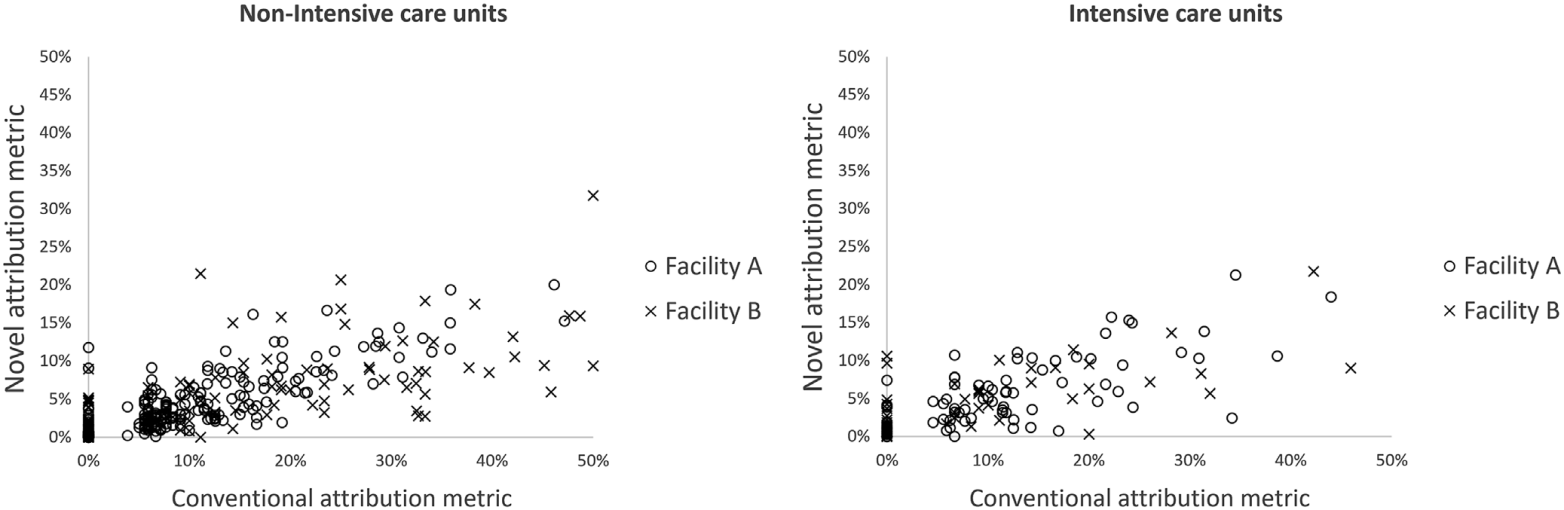
Distribution of the difference in attribution of *Clostridioides difficile* disease when using a novel attribution metric versus a conventional attribution metric, among inpatient intensive care and non-intensive care units.

The sensitivity analysis aggregated the attribution percent measures by quarter rather than monthly. The number of unit-quarter attribution percent data points observed for facility A was 350 (270 non-ICU, 80 ICU) and for facility B was 210 (160 non-ICU, 50 ICU) (Figure 3). The correlation coefficients for non-ICU units were 0.85 (95% CI, 0.82–0.88) and 0.81 (95% CI, 0.77–0.84) and for ICU units were 0.73 (95% CI, 0.60–0.82) and 0.69 (95% CI. 0.51–0.81) at facilities A and B, respectively.

**Figure 4.**
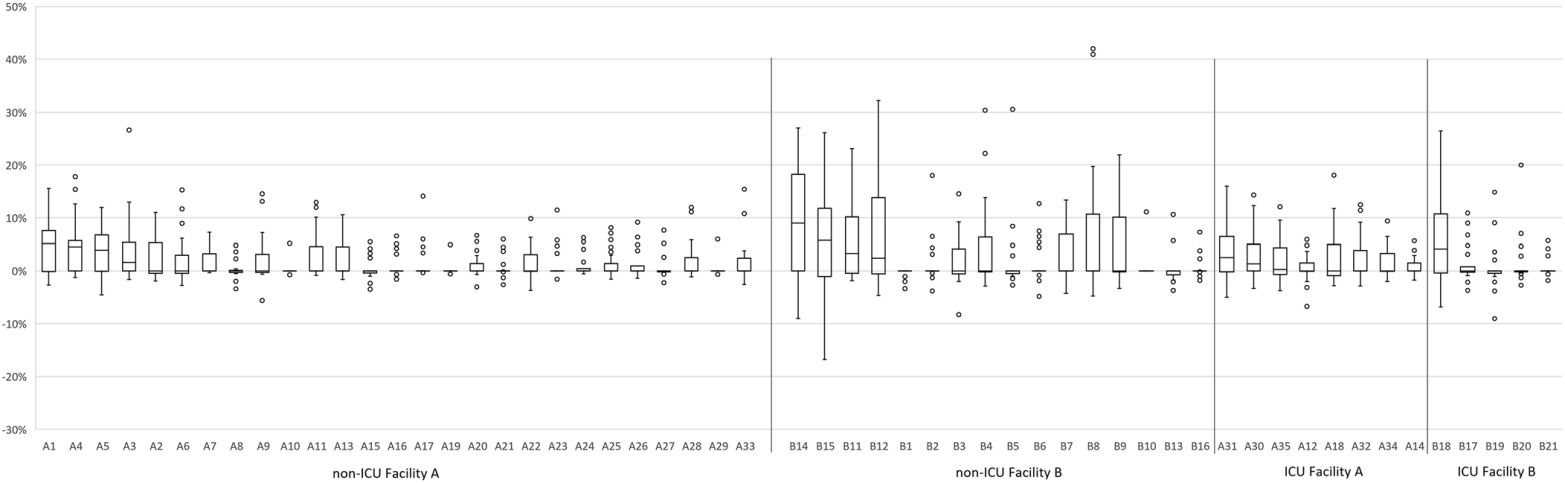
Frequency of the quarterly *Clostridioides difficile* infections attributed to intensive care units and non-intensive care units at two study facilities, comparing conventional and novel attribution metrics.

For all four stratifications (ICU/non-ICU, facility A/B), the distribution of difference in the monthly percent attribution showed higher attribution using NHSN measure than the novel attribution metric: 38% (3/8) of ICU units and 15% (4/27) of non-ICU units in facility A, and 20% (1/5) of ICU units and 25% (4/16) of non-ICU units in facility B had a median difference >0 (Figure 4). The remainder of units had a difference in medians of 0; no units had a difference in medians <0.

In post hoc analyses for facilities A and B, excluding observations with zero attribution by both metrics, 60.2% of observations at facility A and 53.3% of observations at facility B were >0 in both the novel and conventional metrics. Of the remainder, 0.3% and 1.7% of observations, respectively, entailed zero attribution according to the novel metric and >0 attribution by the conventional metric. 39.5% of observations at facility A and 45.0% of observations at facility B had zero attribution according to the conventional metric and >0 attribution using the novel metric (Supplemental material 2, Tables S1 and S2).

## Discussion

In this quality improvement study of the design and initial evaluation of a novel attribution for the source of 727 HAI and 409 non-HAI *C. difficile* infections in two acute care facilities, we found that correlation differed between two methods and attribution shifted to non-acute care settings, including when stratified by facility and unit time. By demonstrating a difference from the conventional metric, this novel metric has the potential to ascribe the preventable risk of *C. difficile* disease more accurately, including acquisition and antibiotic exposure. Subsequent studies will need to validate the accuracy of the attribution, and if validated, the novel metric has the potential to reduce the burden of *C. difficile* disease by more efficiently directing resources preventing transmission and development of disease.

Non-facility attribution, preadmission sources of *C. difficile*, was common in using this novel tool. Our estimates with a 15-day window show that in facility A and B, respectively, 54.2% and 61.9% of attribution days were outside of the index test facility. The value in adding this span of time is to consider other factors which play a role into *C. difficile* infection. Responsibility for *C. difficile* infections is shifted away from study facilities and into locations such as long-term care facilities or into the community, like a patient’s residence. These distributions of sources are roughly comparable in studies demonstrated by authoritative whole-genome-sequenced-based data. One of these studies found 45% of *C. difficile* cases were genetically distinct from each other,^2^ while another found 81%.^6^ In one of the study facilities, whole genome sequencing was completed for *C. difficile* samples. Only 15% of the HAI samples were determined to be genetically related.^7^ Although whole genome sequencing would provide a more conclusive attribution, it would require sequencing all *C. difficile* isolates in a community and still requires establishing an epidemiological linkage between cases. With validation, the attribution metric may be useful until whole genome sequencing is widely available.

NSHN-defined attribution inclines people to prevention measures such as hand hygiene, use of personal protective equipment, and cleaning as the main interventions to stop *C. difficile* transmission. A model found cleaning and screening on admission for *C. difficile* are more effective at preventing transmission,^19^ but hand hygiene and personal protective equipment adherence remains low.^22,23^ This metric may allow Infection Preventionists to focus interventions more efficiently within the facility and potentially outside of acute care facilities. Antibiotic stewardship is an important element which could be refocused after the use of the metric. In our post hoc analysis, 39.5% and 45.0% had zero attribution using the conventional metric and >0 using the novel metric, in facility A and B, respectively. In usual practice, these units would not have accountability for these infections. Unit types that would receive the most scrutiny to increase or redirect efforts are non-ICU units; the “real world” impact of redirecting *C. difficile* reduction efforts on the HAI rates in units identified thusly will be a substantial validation test of the algorithm.

Among our limitations was the 15-day duration of attribution used to estimate the most likely period of acquisition (transmission) and/or antibiotic exposure that precipitated *C. difficile* clinical disease resulting in testing. This estimate is a reasonable approximation given previous reports of incubation period and preceding antibiotic use^10,13,14,24–26^ but will not be a precise estimate for every patient. The study involves only two medical centers with relatively small numbers. The metric would require additional validation in other facilities, larger studies, and using genetic relatedness testing.

In this study, we describe a novel *C. difficile* attribution metric that uses the 14 days preceding the diagnosis and the day of sample collection to provide a potentially more precise estimate of care locations most likely to have transmission risk and antibiotic exposures leading to *C. difficile* disease. If validated, this may prove a cost-effective way to most efficiently and effectively deploy *C. difficile* reduction interventions in healthcare and community settings.

## Supporting information

Doyle et al. supplementary material 1 Doyle et al. supplementary material

Doyle et al. supplementary material 2 Doyle et al. supplementary material

## Supplementary material

For supplementary material accompanying this paper visit https://doi.org/10.1017/ash.2023.516
