# Supplementary material for: A novel approach to correcting attribution of Clostridioides difficile in a healthcare setting
Source: Antimicrob Steward Healthc Epidemiol. 2023 Dec 20;3(1):e246. doi: 10.1017/ash.2023.516 (PMC10753511; doi:10.1017/ash.2023.516)
Supplement: Doyle et al. supplementary material 1 — Doyle et al. supplementary material [file S2732494X23005168sup001.docx]

**Supplemental Material 1: Strengthening the Reporting of Observational Studies in Epidemiology (STROBE) and** **Standards for Quality Improvement Reporting Excellence (SQUIRE) 2.0 Guidelines**

**A Novel Approach to Correcting Attribution of *Clostridioides difficile* in a Healthcare Setting**

|  | **Item No.** | **STROBE items** | **Location** | **SQUIRE items** | **Location** |
| --- | --- | --- | --- | --- | --- |
| **Title and Abstract** | | | | | |
|  | 1 | (a) Indicate the study’s design with a commonly used term in the title or the abstract (b) Provide in the abstract an informative and balanced summary of what was done and what was found | Abstract section | Title: Indicate that the article concerns an initiative to improve healthcare.  Abstract: This is a summary of your work and is the most important section to attract a reader's attention. Please ensure you include a brief background to the problem, the method for your quality improvement project, the overall results and conclusion. | Title line and abstract section |
| **Introduction** | | | | | |
| Background rationale | 2 | Explain the scientific background and rationale for the investigation being reported | Introduction – all paragraphs | Background information about the problem and up-to-date, research and knowledge from the literature. | Introduction – all paragraphs |
| Objectives | 3 | State specific objectives, including any prespecified hypotheses | Introduction – paragraph 4 | Summarize your problem and the focus of your project. | Introduction – paragraph 4 |
| **Methods** | | | | | |
| Study Design | 4 | Present key elements of study design early in the paper | Methods, Data Sources and Outcomes – paragraph 1, 2, Data Visualization – paragraph 1 | Describe any reasons or assumptions that were used to develop the intervention(s) and reasons why you expected them to work. | Not described in the methods |
| Setting | 5 | Describe the setting, locations, and relevant dates, including periods of recruitment, exposure, follow-up, and data collection | Methods, Study Setting – paragraph 1, Data Sources and Outcomes – paragraph 1, 2 |  |  |
| Participants | 6 | *(a) Cohort study* - Give the eligibility criteria, and the sources and methods of selection of participants. Describe methods of follow-up  *Case-control study* - Give the eligibility criteria, and the sources and methods of case ascertainment and control selection. Give the rationale for the choice of cases and controls  *Cross-sectional study* - Give the eligibility criteria, and the sources and methods of selection of participants  *(b) Cohort study* - For matched studies, give matching criteria and number of exposed and unexposed  *Case-control study* - For matched studies, give matching criteria and the number of controls per case | Methods, Data Sources and Outcomes – paragraph 1, 2 |  |  |
| Variables | 7 | Clearly define all outcomes, exposures, predictors, potential confounders, and effect modifiers. Give diagnostic criteria, if applicable. | Methods, Data Sources and Outcomes – paragraph 1, 2 | Explain your strategy for improvement and discuss how you implemented your study. | Methods, Data Sources and Outcomes – paragraph 1, 2, Data Visualization – paragraph 1 |
| Data sources/ measurement | 8 | For each variable of interest, give sources of data and details of methods of assessment (measurement).  Describe comparability of assessment methods if there is more than one group | Methods, Data Sources and Outcomes – paragraph 1, 2 |  |  |
| Bias | 9 | Describe any efforts to address potential sources of bias | Not described in the Methods |  |  |
| Study size | 10 | Explain how the study size was arrived at | Not described |  |  |
| Quantitative variables | 11 | Explain how quantitative variables were handled in the analyses. If applicable, describe which groupings were chosen, and why | Methods, Data Sources and Outcomes – paragraph 1, 2, Data Visualization – paragraph 1, Statistical Methods, paragraph 1, 2 |  |  |
| Statistical methods | 12 | (a) Describe all statistical methods, including those used to control for confounding  (b) Describe any methods used to examine subgroups and interactions  (c) Explain how missing data were addressed  (d) *Cohort study* - If applicable, explain how loss to follow-up was addressed  *Case-control study* - If applicable, explain how matching of cases and controls was addressed  *Cross-sectional study* - If applicable, describe analytical methods taking account of sampling strategy  (e) Describe any sensitivity analyses | Methods, Statistical Methods – paragraph 1, 2 |  |  |
| **Results** | | | | | |
| Participants | 13 | (a) Report the numbers of individuals at each stage of the study (*e.g.*, numbers potentially eligible, examined for eligibility, confirmed eligible, included in the study, completing follow-up, and analyzed)  (b) Give reasons for non-participation at each stage.  (c) Consider use of a flow diagram | Results – paragraph 1, 2 |  |  |
| Descriptive data | 14 | (a) Give characteristics of study participants (*e.g.*, demographic, clinical, social) and information on exposures and potential confounders  (b) Indicate the number of participants with missing data for each variable of interest  (c) *Cohort study* - summarize follow-up time (*e.g.*, average, and total amount) | N/A – there are no participants in the study |  |  |
| Outcome data | 15 | *Cohort study* - Report numbers of outcome events or summary measures over time  *Case-control study* - Report numbers in each exposure category, or summary measures of exposure  *Cross-sectional study* - Report numbers of outcome events or summary measures | Results – paragraph 1, 2, 3 |  |  |
| Main results | 16 | (a) Give unadjusted estimates and, if applicable, confounder-adjusted estimates and their precision (e.g., 95% confidence interval). Make clear which confounders were adjusted for and why they were included  (b) Report category boundaries when continuous variables were categorized  (c) If relevant, consider translating estimates of relative risk into absolute risk for a meaningful time period | Results – paragraph 3 | Provide a summary of what your results showed. Comment on whether there were any unintended consequences such as unexpected benefits, problems, failures, or costs associated with the intervention(s). | Results – paragraph 1, 2, 3, 4, 5 ,6 |
| Other analyses | 17 | Report other analyses done—e.g., analyses of subgroups and interactions, and sensitivity analyses | Results – paragraph 4, 5, 6 |  |  |
| **Discussion** | | | | | |
| Key results | 18 | Summarize key results with reference to study objectives | Discussion – paragraph 1 | Comment on the strengths of the project. Describe any problems you faced and how you navigated these. | Discussion – paragraph 1, 5 |
| Limitations | 19 | Discuss limitations of the study, taking into account sources of potential bias or imprecision. Discuss both direction and magnitude of any potential bias | Discussion – paragraph 4 | Reflect on your project's limitations. | Discussion – paragraph 4 |
| Interpretation | 20 | Give a cautious overall interpretation of results considering objectives, limitations, multiplicity of analyses, results from similar studies, and other relevant evidence | Discussion – paragraph 1, 2, 3, 4, 5 | Describe whether chance, bias, or confounding have affected your results and whether there was any imprecision in the design or analysis of the project. Are more data points required? | Discussion – paragraph 4 |
| Generaliz-  ability | 21 | Discuss the generalizability (external validity) of the study results | Discussion – paragraph 5 | Comment on the limits of generalizability. | Discussion – paragraph 5 |
