# Supplementary material for: A novel approach to correcting attribution of Clostridioides difficile in a healthcare setting
Source: Antimicrob Steward Healthc Epidemiol. 2023 Dec 20;3(1):e246. doi: 10.1017/ash.2023.516 (PMC10753511; doi:10.1017/ash.2023.516)
Supplement: Doyle et al. supplementary material 2 — Doyle et al. supplementary material [file S2732494X23005168sup002.docx]

**SUPPLEMENTAL MATERIAL - A Novel Approach to Correcting Attribution of *Clostridioides* *difficile* in a Healthcare Setting**

**Table of Contents**

[**Supplemental Table S1** 2](#_Toc129866395)

[**Supplemental Table S2** 4](#_Toc129866396)

# **Supplemental Table S1**

| **Unit (30 Months)** non-ICU *ICU* | Percent of highlighted observations (NHSN, Novel) | | | | |
| --- | --- | --- | --- | --- | --- |
|  | 0,0 = exclude | 0,X | Sum of {X,0} and {X,X} | X,0 | X,X |
| A10 | (24) | **83.3%** (5) | 16.7% (1) | (0) | (1) |
| A29 | (25) | **80.0%** (4) | 20.0% (1) | (0) | (1) |
| A27 | (14) | **75.0%** (12) | 25.0% (4) | (0) | (4) |
| A15 | (15) | **66.7%** (10) | 33.3% (5) | (0) | (5) |
| A19 | (27) | **66.7%** (2) | 33.3% (1) | (0) | (1) |
| A8 | (10) | **65.0%** (13) | 35.0% (7) | (0) | (7) |
| A6 | (8) | **54.5%** (12) | 45.5% (10) | (0) | (10) |
| A2 | (6) | **54.2%**(13) | 45.8% (11) | (0) | (11) |
| A9 | (8) | **50.0%** (11) | 50.0% (11) | (0) | (11) |
| *A14* | (15) | **46.7%** (7) | 53.3% (8) | (0) | (8) |
| A16 | (19) | **45.5%** (5) | 54.5% (6) | (0) | (6) |
| A21 | (19) | **45.5%** (5) | 54.5% (6) | (1) | (5) |
| A22 | (12) | **44.4%** (8) | 55.6% (10) | (0) | (10) |
| A28 | (14) | **43.8%** (7) | 56.2% (9) | (0) | (9) |
| *A12* | (14) | **43.8%** (7) | 56.2% (9) | (0) | (9) |
| A17 | (23) | **42.9%** (3) | 57.1% (4) | (0) | (4) |
| A25 | (16) | **42.9%** (6) | 57.1% (8) | (0) | (8) |
| *A35* | (4) | **42.3%** (11) | 57.7% (15) | (0) | (15) |
| *A34* | (11) | **42.1%** (8) | 57.9% (11) | (0) | (11) |
| A20 | (15) | **40.0%** (6) | 60.0% (9) | (0) | (9) |
| *A18* | (7) | **39.1%** (9) | 60.9% (14) | (0) | (14) |
| A24 | (19) | **36.4%** (4) | 63.6% (7) | (0) | (7) |
| *A32* | (15) | **33.3%** (5) | 66.7% (10) | (0) | (10) |
| A13 | (14) | **31.2%** (5) | 68.8% (11) | (0) | (11) |
| A3 | (8) | **27.3%** (6) | 72.7% (16) | (0) | (16) |
| A11 | (15) | **26.7%** (4) | 73.3% (11) | (0) | (11) |
| A7 | (18) | **25.0%** (3) | 75.0% (9) | (0) | (9) |
| *A31* | (2) | **25.0%** (7) | 75.0% (21) | (0) | (21) |
| A5 | (1) | **24.1%** (7) | 75.9% (22) | (0) | (22) |
| A4 | (5) | **24.0%** (6) | 76.0% (19) | (0) | (19) |
| A26 | (21) | **22.2%**(2) | 77.8% (7) | (0) | (7) |
| A1 | (2) | **21.4%**(6) | 78.6% (22) | (0) | (22) |
| A33 | (20) | **20.0% (**2) | 80.0% (8) | (0) | (8) |
| *A30* | (11) | **15.8%** (3) | 84.2% (16) | (1) | (15) |
| A23 | (23) | **14.3%** (1) | 85.7% (6) | (0) | (6) |
| **Total** |  | **225 (39.5%)** | **345 (60.5%)** | **2 (0.3%)** | **343 (60.2%)** |

# **Supplemental Table S2**

| **Unit (30 Months)** non-ICU *ICU* | Percent of highlighted observations (NHSN, Novel) | | | | |
| --- | --- | --- | --- | --- | --- |
|  | 0,0 = exclude | 0,X | Sum of {X,0} and {X,X} | X,0 | X,X |
| B1 | (26) | **100.0%**(4) | 0.0% (0) | (0) | (0) |
| B13 | (14) | **87.5%** (14) | 12.5% (2) | (0) | (2) |
| B5 | (10) | **70.0%** (14) | 30.0% (6) | (0) | (6) |
| B16 | (21) | **66.7%** (6) | 33.3% (3) | (0) | (3) |
| *B19* | (14) | **62.5%** (10) | 37.5% (6) | (0) | (6) |
| *B20* | (16) | **57.1%** (8) | 42.9% (6) | (1) | (5) |
| *B21* | (23) | **57.1%** (4) | 42.9% (3) | (0) | (3) |
| *B17* | (14) | **56.3%** (9) | 43.7% (7) | (0) | (7) |
| B2 | (19) | **54.5%**(6) | 45.5% (5) | (0) | (5) |
| B3 | (12) | **50.0%** (9) | 50.0% (9) | (0) | (9) |
| B6 | (19) | **45.5%** (5) | 54.5% (6) | (0) | (6) |
| B9 | (5) | **44.0%** (11) | 56.0% (14) | (0) | (14) |
| B4 | (11) | **42.1%** (8) | 57.9% (11) | (0) | (11) |
| B12 | (3) | **40.7%** (11) | 59.3% (16) | (1) | (15) |
| *B18* | (2) | **39.3%** (11) | 60.7% (17) | (1) | (16) |
| B7 | (14) | **31.2%** (5) | 68.8% (11) | (0) | (11) |
| B15 | (1) | **31.0%** (9) | 69.0% (20) | (0) | (20) |
| B8 | (11) | **26.3% (**5) | 73.7% (14) | (0) | (14) |
| B11 | (9) | **23.8%** (5) | 76.2% (16) | (2) | (14) |
| B14 | (4) | **19.2%** (5) | 80.8% (21) | (0) | (21) |
| B10 | (29) | **0.0%** (0) | 100.0% (1) | (1) | (0) |
| **Total** |  | **159 (45.0%)** | **194 (55.0%)** | **6 (1.7%)** | **188 (53.3%)** |
